# Identification of transcription factor contexts in literature using machine learning approaches

**DOI:** 10.1186/1471-2105-9-S3-S11

**Published:** 2008-04-11

**Authors:** Hui Yang, Goran Nenadic, John A Keane

**Affiliations:** 1 School of Computer Science, University of Manchester, Manchester, UK

## Abstract

**Background:**

Availability of information about transcription factors (TFs) is crucial for genome biology, as TFs play a central role in the regulation of gene expression. While manual literature curation is expensive and labour intensive, the development of semi-automated text mining support is hindered by unavailability of training data. There have been no studies on how existing data sources (e.g. TF-related data from the MeSH thesaurus and GO ontology) or potentially noisy example data (e.g. protein-protein interaction, PPI) could be used to provide training data for identification of TF-contexts in literature.

**Results:**

In this paper we describe a text-classification system designed to automatically recognise contexts related to transcription factors in literature. A learning model is based on a set of biological features (e.g. protein and gene names, interaction words, other biological terms) that are deemed relevant for the task. We have exploited background knowledge from existing biological resources (MeSH and GO) to engineer such features. Weak and noisy training datasets have been collected from descriptions of TF-related concepts in MeSH and GO, PPI data and data representing non-protein-function descriptions. Three machine-learning methods are investigated, along with a vote-based merging of individual approaches and/or different training datasets. The system achieved highly encouraging results, with most classifiers achieving an F-measure above 90%.

**Conclusions:**

The experimental results have shown that the proposed model can be used for identification of TF-related contexts (i.e. sentences) with high accuracy, with a significantly reduced set of features when compared to traditional bag-of-words approach. The results of considering existing PPI data suggest that there is not as high similarity between TF and PPI contexts as we have expected. We have also shown that existing knowledge sources are useful both for feature engineering and for obtaining noisy positive training data.

## Background

Over the past decade, text mining techniques have been used to support the (semi-)automatic extraction of information from biomedical literature. A number of systems have been designed to capture information on general biological molecular interactions [1-9] or interactions focused on a particular organism of interest (such as Homo sapiens [10], Drosophila melanogaster [11], and Saccharomyces cerevisiae [12]). In addition, specific types of molecular interactions have been targeted (e.g. inhibition relationships between biological entities [13], or enzyme and metabolic pathways [14]). Several evaluation challenges and exercises have been organised to assess the development in the field, in particular for protein-protein interactions (PPI) (e.g. BioCreative [15], LLL05 Challenge [16], etc.).

A topic that has been of particular interest in biomedicine is the investigation of gene regulatory networks, in which transcription factors play a crucial role. A transcription factor (TF) is a protein that regulates binding of RNA polymerase and initiation of transcription [17]. TFs are regulators of gene expression and influence almost all biological processes in an organism. Existing TF databases (such as TRANSFAC [18], FlyBase [19], ORegAnno [20]) are largely based on manual literature curation. Despite their importance for genome biology, curation of these databases is far from satisfactory for many organisms, partially due to the difficulties in locating the information linked to transcription regulation stored in an ever increasing volume of relevant literature.

In this paper we investigate the automatic extraction of TF-related contexts (at the sentence level) to support curation of transcription factors from biomedical literature. To the best of our knowledge, our work is one of the first attempts to apply text-mining techniques to the task [21]. As opposed to PPI contexts (representing interactions between proteins), our aim is to locate a specific type of interactions related to gene regulation by TFs. More precisely, we are focused on a specific *role* of certain biological entities: our targets are contexts that mention special proteins (i.e. transcription factors) that regulate gene expressions. The following is a typical example of a TF-related context:

… Reconstituted in vitro transcription reactions and deoxyribonuclease I footprinting assays confirmed the ability of TRF1 to bind preferentially and direct transcription of the tudor gene from an alternate promoter…

Several actors and events (e.g. proteins, DNA, transcriptions, DNA binding) can be typically found in such contexts (see Table 1). One of the most important features of TFs is transcription regulation where transcription factors *interact* with other regulatory *proteins* to either increase or decrease the transcription of specific genes. Thus, transcription regulation contexts could be regarded as a type of PPI context and in this paper we further investigate the degree of similarities between them.

**Table 1:** Examples of typical actors and events in transcription factor contexts

**Actor, event**	**Examples**
***DNA binding***	*DNA binding; DNA binding protein; DNA binding region DNA binding property, DNA binding affinity; DNA binding specificity*
***Transcription***	*transcription; transcriptional regulator; gene transcription; transcription repression; transcription reaction; transcription activity*
***Protein actor***	*transcription factor; protein factor; transcription repressor transcriptional activator; transcriptional mediator; heterodimer*
***DNA actor***	*enhancer; promoter; reporter*

We focus on machine learning (ML) approaches and discuss creation of suitable training datasets that can support the task. More specifically, we present a series of investigations and experiments that aim to clarify the following issues:

• **Training data:** can we use existing knowledge bases (e.g. the MeSH thesaurus [22] and GO ontology [23]) to create a collection of noisy but useful positive data? Would it be feasible to use PPI contexts to support TF-curation?

• **Features:** is a small set of biological features (e.g. gene and protein names, TF-specific terms, interaction verbs, etc.), which are believed to be representative of transcription factors, enough to identify TF-related sentences?

• **Machine learning**: which techniques are effective for the extraction of TF-related contexts?

In the following section we present the methods and resources that have been used in our investigations. After presenting the experiments and results, we compare our approach to related work in the domain and give some conclusions and directions for future work.

## Methods

**Figure 1:**
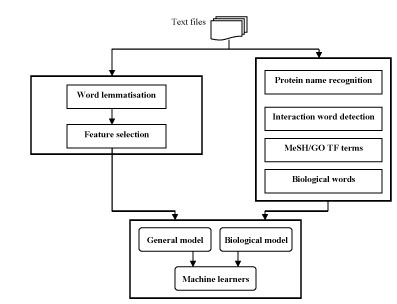
Overall architecture of the approach.

We approached the problem of extracting TF-related contexts as a binary classification task: given a sentence, we aim to classify it as TF-related (positive) or not (negative). We consider three major components: selection of relevant features to support classification, obtaining training data to build classifiers, and selection of ML approaches to be employed for context recognition. We have analysed two types of features: in the *generic* model (GM), we follow the standard bag-of-words approach; in the *biological* model (BM), we consider only features that reflect the biological profile of the task. Three different machine learning algorithms are applied to train TF classifiers based on the two learning models. The overall approach is presented in Fig. 1.

### Feature engineering

In the generic, word-based model (GM), standard word lemmatisation (using GeniaTagger [24]) is employed along with a feature selection procedure to reduce the feature space. We used Pearson's chi-square (χ^2^) test [25] to rank the words in the descending order of their likelihoods of distinguishing the class. The threshold τ of chi-square statistics used for feature selection is calculated using the following equation:

$$\tau =\frac{\sum_{w}{\left(f_{ow}-f_{ew}\right)}^{2}/f_{ew}}{∥w∥}$$

where *f_ow_* denotes the frequency of the observed word *w* and *f_ew_* is the frequency of the expected values; ‖*w*‖ is the total number of the words in the collection. A sentence vector is built by using the features above the threshold τ for all words that are present in it.

In the biological model (BM), the following features are identified in candidate sentences: gene/protein names, interaction words, TF-related MeSH and GO terms, and other biological words. Our rationale was simple: target sentences generally describe *interactions* between TFs (proteins) and target genes, and thus we expect that gene/protein names are important features as are the interaction words [26]. Protein/gene names are recognised by combining the outputs from two publicly available gene name taggers, ABNER [27] and LingPipe [28] (the integrated results achieved F-measure of 78.6% on average). A thesaurus containing interaction words has been collected from the TF and PPI data (mentioned below). All morphological and derivational variants (e.g. *regulate*, *regulation*, *regulatory*) have been included, resulting in 391 potential interaction-word form features.

MeSH and GO terms related to transcription regulation are also considered as potentially important features and have been collected from these two resources, resulting in 247 MeSH terms (subheading ‘*Transcription Factor*’ and its descendents) and 223 TF-related GO terms (based on the TF-related term list curated by [29], which has been extended by all their descendents). Moreover, we have constructed a dictionary of biologically relevant words by tokenising all the terms contained in the MeSH thesaurus and the GO ontology (not only TF-related terms). After removing stop-words (using the SMART system's stopword list of 524 common words [30]) and discarding words with fewer than 3 characters, the dictionary contains around 50,000 words, which have been used as potential features in the BM model.

A feature vector for a given sentence in the BM model contains the following features. First, for each word from the biological dictionary that is present in the sentence, a feature is added (***biological-word*** features), as well as for each interaction word that occurs in the sentence but is not contained in the biological word dictionary (***interaction-word*** features). Then, the following binary features are generated:

- ***has-protein*** – flagged if the sentence contains at least one protein/gene name;

- ***has-two-proteins*** – flagged if the sentence contains at least two unique protein/gene names;

- ***has-interaction-word*** – flagged if the sentence contains at least one interaction word;

- ***has-two-interaction-words*** – flagged if the sentence contains at least two unique interaction words;

- ***has-MeSH-TF-term*** – flagged if the sentence contains at least one MeSH TF term;

- ***has-two-MeSH-TF-terms*** – flagged if the sentence contains at least two unique MeSH TF terms;

- ***has-GO-TF-term*** – flagged if the sentence contains at least one GO TF term;

- ***has-two-GO-TF-terms*** – flagged if the sentence contained at least two unique GO TF terms.

These feature vectors are used in three different machine learning algorithms (Naive Bayes (NB), Support Vector Machine (SVM), and Maximum Entropy (ME)) to learn the classifiers.

### Building training and testing datasets

Building a training set for the extraction of TF sentences proved to be the most difficult and time consuming step. The only suitable and publicly available source is the FlyTF database (the Drosophila Transcription Factor database [31]). This is a manually curated database that contains transcription information based on FlyBase/GO annotation data and the DBD Transcription Factor Database [32]. Some of the records in the database are supported by “traceable author statements”, including sentences from the literature. We have extracted 491 sentences from the database, which seemed as not being enough for a larger scale investigation on retrieving TF-related sentences. We have therefore considered additional sources to support the task by obtaining noisy and weak positive and negative training data.

#### Non-Protein-Function Description (NonPF) data

We used negative sentences from the Prodisen corpus [33], which has been constructed for functional descriptions of genes and proteins, as negative data. A total of 1700 sentences that have been marked as “not gene function description” are randomly collected from the corpus for training and testing.

#### MeSH and GO TF-related descriptions

As mentioned above (cf. feature engineering), both the MeSH and GO databases contain TF-related concepts. MeSH terms located under the subheading ‘*Transcription Factor*’ describe various types of transcription factor concepts which are classified according to either their structure of DNA-binding domains or their regulatory function. In addition, GO annotation information is usually used as a main source for the curation and exploration in transcription factor databases such as FlyTF and TFDB [29]. We have therefore collected *definitions* of TF-related terms from the MeSH and GO databases to create a *noisy* positive set of TF-related sentences. In addition to sentences in definitions, synonym lists are treated as TF-related sentences. Together with FlyTF data, we have collected around 1700 positive sentences (referred to as *TF data*).

The suitability of TF-related definitions from the MeSH thesaurus and GO database as positive data has been tested on the existing FlyTF data. We performed a separate experiment (details are listed in the Experiment section) in which only the MeSH and GO TF data was used for training, while the FlyTF data was used as the test data to evaluate the performance. Generally, the precision was well above 90% with the average recall around 70%, which supported our assumption that this data can be used for learning.

#### PPI data

There have been extensive work and several resources available for PPI-focused text mining systems (see related work discussed later). The reason for us to consider PPI data is due to a potential functional similarity between transcriptional regulation (where transcription factors interact with other regulatory proteins to either increase or decrease the transcription of specific genes) and generic PPI contexts. The aim was to investigate the possibility of using PPI data as training data for TF classification. Our rationale was the following: if PPI and TF contexts are indeed similar, then it would be difficult to differentiate between the two, and a (good) TF-classifier would generally achieve a lower precision on a dataset that contains both TF and PPI examples. On the other hand, if these two context types are generally different, then it would be easier to construct a classifier that performs well on TF and PPI data. We have tested this hypothesis by using PPI data as *negative* data and comparing it to the results obtain by using real negative data (NonPF). If PPI data can indeed be used as negative examples, then we would expect at least the same precision as for the NonPF (negative) data. To generalise the concept of PPI, the data has been collected from various sources including the datasets for LLL05 Challenge [16], BioCreAtIvE-PPI Corpus compiled by J. Hakenberg [34], PICorpus [35] and GeneRIF HIV Interaction Corpus [36].

To summarise the data preparation step, the data used for TF-sentence classification is organised into three different sets of contexts, namely, TF data (including FlyTF, MeSH and GO TF-related sentences, used as positive examples), non-protein-function-description (NonPF) and protein-protein interaction (PPI) data. The NonPF and PPI datasets are separately treated as *negative* and *noisy negative* data to constitute two experimental settings: TF&NonPF and TF&PPI. The three data collections have been prepared at the sentence level, and they all have a similar number of sentences (around 1700 each).

## Experiments and results

The detailed statistics for the datasets used in the experiments are given in Table 2. Table 3 presents the details of the features generated for each of the datasets after the feature selection process (using chi-square statistics).

**Table 2:** Statistics for the datasets used in the experiments

	**TF data (positive data)**			**PPI data (noisy negative data)**				**NonPF data (negative data)**
	
	**FlyTF**	**MeSH**	**GO**	**LLL**	**BioCreAtIvE**	**PICorpus**	**GeneRIF HIV**	**Prodisen**
**# sentences per resource**	491	712	477	77	283	127	1200	1700
**total # sentences**	1680			1687				1700

**Table 3:** Feature statistics for different datasets (GM = generic model; BM = biological model). Note that the feature list used in the BM model is longer than that of the GM model due to the additional binary biological features (*has-protein*, *has-two-proteins*, etc.).

		**TF data**	**PPI Data**	**NonPF Data**
**total # features**	**GM**	1327	1188	1780
	**BM**	803	760	1306
**# features per sentence**	**GM**	9.70	14.44	11.43
	**BM**	12.87	17.73	9.78

Before presenting the results of the identification of TF-related sentences, we first report our findings on the usefulness of TF-related data collected from the MeSH and GO databases as positive data for the task. We also present an analysis of the similarities between TF and PPI data. In all experiments, the performance has been evaluated using 5-fold cross-validation (train on 80% and test on 20%, repeated 5 times on a different 20% each time), by using precision (*P*), recall (*R*) and F-measure (*F*) metrics defined as follows:

$$R\;=\;\frac{TP}{TP\;+\;FN}\;\;\;\;\;\;\;\;\;\;\;\;\;\;\;\;\;\;\;\;\;\;\;\;P=\frac{TP}{TP+FP}\;\;\;\;\;\;\;\;\;\;\;\;\;\;\;\;\;\;\;\;\;F-measure=\frac{2PR}{P+R}$$

where *TP* (true positive) is the number of correctly recognised TF sentences, *FN* (false negative) is the number of TF sentences not identified by the system, and *FP* (False Positive) the number of TF sentences that are incorrectly detected. For most experiments we compare the results obtained from the two learning models (generic and biological) and three ML approaches (SVM, NB, ME). The SVM classifier was built with the TinySVM package [37] using the polynomial kernel, and the NB and ME classifiers were implemented with MALLET [38] with the default parameters.

### Suitability of MeSH and GO TF-related data as positive examples

As described earlier, we hypothesised that the descriptions of TF-related terms from the MeSH and GO databases could be used for detecting TF-related sentences. To verify this hypothesis, we used this data as the *noisy positive* examples for learning (with NonPF and PPI as negative examples) and the FlyTF data (real positive examples) as exclusive positive data for testing. Table 4 shows the performance of the three machine-learning classifiers.

**Table 4:** Performance of the three machine-learning classifiers on the FlyTF test data using only MeSH and GO TF data as positive training data (GM = generic model; BM = biological model)

		**SVM**			**NB**			**ME**		
		
		**P**	**R**	**F**	**P**	**R**	**F**	**P**	**R**	**F**
**MeSH+GO & NonPF**	**GM**	.9328	.7352	.8223	.9477	.8859	.9158	.9595	.7230	.8246
	**BM**	.9542	.7210	.8213	.9595	.8676	.9112	.9802	.7047	.8199
**MeSH+GO & PPI**	**GM**	1.000	.6986	.8225	1.000	.6354	.7771	.9972	.7210	.8369
	**BM**	.9810	.6314	.7683	1.000	.5234	.6872	.9816	.6517	.7834

The results show that the precision achieved was well above 90% on both datasets (MeSH+GO&NonPF, MeSH+GO&PPI), suggesting that the TF-related term definitions from the MeSH thesaurus and GO database – despite being noisy positive data – are suitable for capturing features for TF-sentence classification. The relatively lower recall results (52-72%) reflect the issue that this data – although accurate – does not cover all expressional variations used in TF sentences. To demonstrate potential usefulness of (real positive) data from FlyTF for recall, we have conducted a set of experiments in which we added 80% FlyTF data to the MeSH+GO training (positive) data, and 20% FlyTF data was left for testing (5-fold cross-validation was used). Table 5 shows the effects of adding the FlyTF data to the training data: there was a substantial increase in recall (10-20%) and accordingly in F-measure (with a limited drop in precision, only for TF&NonPF data).

**Table 5:** Performance of the three machine-learning classifiers on the FlyTF test data using both MeSH and GO TF data and part of the FlyTF data as positive training data (GM = generic model; BM = biological model)

		**SVM**			**NB**			**ME**		
		
		**P**	**R**	**F**	**P**	**R**	**F**	**P**	**R**	**F**
**TF & NonPF**	**GM**	.9271	.8910	.9087	.9308	.9592	.9447	.9588	.8533	.9029
	**BM**	.9455	.8925	.9182	.9527	.9450	.9488	.9770	.8655	.9109
**TF & PPI**	**GM**	1.000	.9183	.9574	1.000	.8879	.9406	1.000	.9124	.9541
	**BM**	.9936	.8926	.9403	1.000	.8370	.9112	.9885	.8818	.9321

### Similarities between TF and PPI contexts

The last point made above was a surprise: when the PPI data was used as negative examples for training, the precision was overall better than when the NonPF data was used (see tables 4 and 5). This suggests that PPI data seems to better discriminate TF contexts than the NonPF dataset. High precision (for each of the three classifiers) suggests that TF and PPI contexts are not as similar as expected, implying that PPI data could provide promising *noisy negative* data for learning TF classifiers. Furthermore, we calculated feature distribution differences between the TF and PPI datasets, and also between the TF and NonPF data, using the Average Kullback-Leibler (AKL) divergence [39]. For two datasets *q* and *p*, the AKL divergence is calculated as:

$$AKL\left(q,p\right)=\frac{1}{2}\sum_{x}\left(q\left(x\right)\log\frac{q\left(x\right)}{p\left(x\right)}+p\left(x\right)\log\frac{p\left(x\right)}{q\left(x\right)}\right)$$

**Figure 2:**
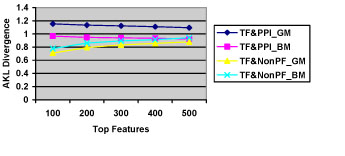
The average KL divergence of feature distributions between (1) TF and PPI, and (2) TF and NonPF datasets for the GM and BM models, when the top ranked features are considered (TF& PPI_GM = feature distribution in TF vs. feature distribution in PPI in GM model, etc.)

Here, *q*(*x*) and *p*(*x*) are occurrence probabilities of the feature x in datasets *q* and *p*, respectively. In our case, feature probabilities are calculated using the chi-square statistics value of each feature in the collection. The divergence results for TF/PPI and TF/NonPF datasets with the two feature models (GM and BM) are presented in Fig. 2, with various numbers of top ranked features selected from the datasets. Overall, the divergence between the TF and PPI data was much larger than that of TF and NonPF data. This partly explains why the accuracy on the TF&PPI dataset generally outperformed that of TF&NonPF.

Obviously, despite the high precision in discriminating between generic PPI and TF contexts, there are PPI sentences that are also TF contexts. Table 6 presents “confusion” examples of PPI sentences wrongly classified as TF-contexts, and TF-sentences marked as non-TF (i.e. PPI) contexts. For example, sentences containing ‘*transcription*’ are usually *correctly* identified as (positive) TF contexts, while, on the other hand, some TF sentences, which do not contain strong TF discriminative features, are wrongly recognised as PPI examples. Still, the results for the TF&PPI dataset were encouraging and we decided to conduct further experiments with the PPI data used as (noisy) negative data (in addition to the NonPF data).

**Table 6:** Examples of confused contexts in the TF & PPI dataset

**Correct**	**Predicted**	**Example**
PPI	TF	*Transcription Factor IIH (TFIIH) and p300 act cooperatively to enhance Vpr effects on glucocorticoid receptor transactivation*.
PPI	TF	*These studies show that VES induces growth inhibition of BT-20 cells through a mechanism that involves cyclin A-negative regulation of E2F-mediated transcription*.
PPI	TF	*Adenovirus E1A protein represses activation by Vpr by competing for binding to p300, suggesting that p300 is required for activation of HIV transcription by Vpr*.
TF	PPI	*It plays a role in HOMEOSTASIS of GLUCOSE and controls expression of GLUT2 PROTEIN*.
TF	PPI	*Mutations in hepatocyte nuclear factor 1-beta are associated with renal CYSTS and MATURITY-ONSET DIABETES MELLITUS type 5*.

### Performance comparisons for the TF-sentence classification task

**Figure 3:**
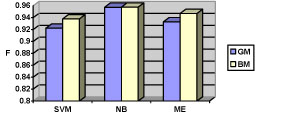
The F-measures of the three machining learning approaches on the TF&NonPF dataset (GM = generic model; BM = biological model)

**Figure 4:**
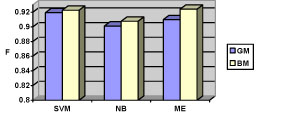
The F-measure of the three machining learning approaches on the TF&PPI dataset (GM = generic model; BM = biological model)

After the preliminary experiments, the two datasets (TF&NonPF and TF&PPI) were used to train three machine-learning classifiers (SVM, NB, ME), using 5-fold cross-validation. Table 7 and figures 3 and 4 present the results, while a detailed discussion is given below.

**Table 7:** Performance of the three machine-learning classifiers on the TF & NonPF and TF & PPI datasets using 5-fold cross-validation (GM = generic model; BM = biological model)

		**SVM**			**NB**			**ME**		
		
		**P**	**R**	**F**	**P**	**R**	**F**	**P**	**R**	**F**
**TF & NonPF**	**GM**	.9342	.9104	.9222	.9413	.9744	.9576	.9638	.9042	.9330
	**BM**	.9421	.9343	.9380	.9434	.9726	.9578	.9591	.9351	.9470
**TF & PPI**	**GM**	.8938	.9463	.9193	.8767	.9268	.9010	.8685	.9554	.9099
	**BM**	.9092	.9367	.9227	.8892	.9268	.9076	.8974	.9524	.9241

#### Comparison of the feature models (GM vs. BM)

The biological model consistently out-performed the generic model on both TF&NonPF and TF&PPI datasets. The experimental results show that the performance of individual classifiers improved up to 2.5%, while being achieved with fewer features (recall Table 3: the BM feature sets were almost one third of the GM model). Although the biological model requires additional pre-processing for feature extraction (e.g. gene name identification), this is typically a step in a typical text mining pipeline that would be beneficial for other tasks as well. Overall, the results suggest that biological features (gene/protein names, interaction words, MeSH/GO TF terms) seem to be to some extent more useful than non-biological features for TF-sentence identification. Still, in some cases, the BM model achieved only less than 1% improvement on the F-measure compared to the GM model. One explanation for such a modest improvement is a potential overlap between BM and GM features. We explored the top 350 features (measured by chi-square statistics) from the GM and BM models used in the TF&NonPF dataset, and found that only 9.4% features of the GM model (33 features) has not appeared in the BM feature list. This implies that the best features for classification are indeed biological words, which have been selected by both models.

#### Using more negative data for training

To ensure unbiased learning of the classifiers, in the first set of experiments (Table 7) we have deliberately constructed the training datasets with balanced numbers of positive and negative examples. However, in a real-world setting, it seems that non-relevant TF contexts are far more frequent than relevant ones. To investigate the impact of an unbalanced but more realistic training dataset containing more negative cases, we performed another set of experiments with additional 1200 PPI sentences and 1000 NonPF sentences added to the corresponding (negative) training data and examined the performance of the classifiers on the unchanged test data. The results presented in Table 8 show just a marginal improvement when compared to the balanced-training data scenario (slightly improved accuracy, with a small drop in the recall).

**Table 8:** Performance of the three machine-learning classifiers on the TF & NonPF and TF & PPI datasets with additional negative examples for training using 5-fold cross-validation (GM = generic model; BM = biological model)

		**SVM**			**NB**			**ME**		
		
		**P**	**R**	**F**	**P**	**R**	**F**	**P**	**R**	**F**
**TF & NonPF**	**GM**	.9592	.8967	.9269	.9472	.9708	.9588	.9700	.8863	.9263
	**BM**	.9602	.9242	.9418	.9371	.9661	.9513	.9609	.9208	.9404
**TF & PPI**	**GM**	.8959	.9469	.9207	.8743	.9149	.8941	.8760	.9542	.9134
	**BM**	.9103	.9379	.9239	.8891	.9119	.9004	.9058	.9506	.9277

#### Comparison of ML approaches

Tables 7 and 8 show that the three ML approaches obtained a high precision (generally over 90%), suggesting that TF contexts contain distinguished features which provide strong discriminating power. Still, performance of the classifiers was not consistent on the two datasets. The NB classifier excelled the other two classifiers on the TF&NonPF dataset with an F-measure of over 95% on average, but it performed worse on the TF&PPI dataset (F-measure dropped down below 91%). The SVM classifier was the best on the TF&PPI dataset, but on the TF&NonPF dataset it did not work very well, especially for the generic model. The inconsistent performance of the NB and SVM classifiers (the ME classifier was more stable) can be partially explained by the differences between feature distributions in two datasets (see Fig. 2 for the AKL divergence).

### Merging results from different classifiers

The inconsistent results obtained by different classifiers prompted us to analyse the results obtained by combining their outputs. We investigated a vote-based merging through two stages: first, the outputs from three different classifiers trained on the *same dataset* are combined together according to different voting strategies (Stage I); then, the results integrated from the *distinct training datasets* (TF&NonPF, TF&PPI) are merged together to form the final classification results (Stage II).

#### Stage I: merging results from the classifiers trained on the same dataset

We experimented with the biological model only. Three voting approaches have been applied: *unanimous* (i.e. all vote), *any* (i.e. any vote) or *majority* (at least 2 out of 3 votes). Table 9 shows the performance of Stage I. It is a no surprise that the unanimous voting strategy improved precision, while the voting based on positive outcome from any classifier improved the overall recall performance. However, the best merged F-measure was achieved by the majority voting method, with a marginally worse performance compared to the best single classifier. It is reasonable to expect that the majority voting has a slightly lower F-measure as it only builds the results by agreeing on the judgment from the majority.

**Table 9:** Stage I performance, after the result merging from the three different classifiers learned on the same dataset (using the biological model), along with the best performance in each column before and after Stage I highlighted

		**TF & NonPF data**			**TF & PPI data**		
		
		**P**	**R**	**F**	**P**	**R**	**F**
**before Stage I**	**SVM**	.9381	.9420	**.9039**	.9488	**.9258**	
	**NB**	.9315	**.9720**	**.9514**	.8787	.9143	.8961
	**ME**	.9327	.9474	.8895	**.9536**	.9204	
**after Stage I**	**2/3 majority**	.9576	.9427	.8966	.9515	**.9236**	
	**unanimous**	**.9748**	.9242	**.9279**	.8836	.9052	
	**any**	.9141	**.9785**	.9452	.8598	**.9815**	.9166

#### Stage II: merging results from the classifiers trained on different datasets

It is obvious that the classifiers learned on different datasets may rely on different classification features. By merging the results from different datasets, we investigated potential complementarities. Two types of result filtering were considered: *unanimous voting* and *any voting*. Note that each time the results from two training datasets to be merged are obtained using the same voting strategy at Stage I.

The final merged results are reported in Table 10. The best precision, recall, and F-measure generated in Stage II basically outperformed the results produced at Stage I as well as those from the individual classifiers. The best two F-measure values with most balanced precision and recall were obtained using a combination strategy with the 2/3 majority voting (Stage I) and any voting (Stage II), and the one with the any voting (Stage I) plus unanimous voting (Stage II). The former method achieved F-measure of 97.69% with a high recall (99.46%), while F-measure in the latter reached as high as 97.93% with a ‘perfect’ precision (100%). These results confirm our hypothesis on a complementary relation between the results obtained from the TF&PPI and TF& NonPF data sources. This means that the result merging method could be an effective approach for performance improvement through different contributions from the two training datasets.

**Table 10:** Stage II performance, after combining the results from the two datasets (TF & NonPF and TF & PPI); the best combination results are highlighted

		**Stage I**								
		
		**2/3 majority**			**unanimous**			**any**		
		
		**P**	**R**	**F**	**P**	**R**	**F**	**P**	**R**	**F**
**Stage**	**unanimous**	**1.000**	.9036	.9493	**1.000**	.8339	.9094	**1.000**	.9595	**.9793**
**II**	**any**	.9598	**.9946**	**.9769**	.9748	**.9685**	**.9716**	.9115	**.9994**	.9534

## Related work

Approaches to the extraction of protein-protein interactions and other biological relationships from biomedical text vary widely. Previous research efforts have generally focused on either statistical methods (e.g. co-occurrence of biological entities like protein names or word frequency information [7,40,41]), or linguistics approaches including shallow and deep parsing, applying simple pattern- or rule-based matching [4,42] or complex template- or frame-based processing [9,43-45]. In addition, a number of research projects have relied on machine learning. For example, Donaldson and colleagues [46] built a prototype system to populate a knowledge base with PPI data recognised by an SVM classifier. Jansen and associates [47] reported on a Bayesian network to predict PPI in yeast. Sugiyama and colleagues [48] investigated several machine learning techniques, such as K-nearest neighbour rule, decision tree, neural network, and SVM, to verify the effectiveness of ML approaches in detecting PPI.

Similarly to other ML-approaches, we have employed different machine methods (naive Bayes, SVM, and Maximum Entropy) to discover contexts describing transcription factors. However, our system differs from the related work in the following aspects:

(1) Our approach is focused on a specific *role* of certain biological entities: our targets are special proteins (i.e. transcription factors) that regulate gene expressions. Due to the particular role that TFs have in gene regulation, the objective of our system is to detect relevant text contexts related to this specific biological *function* and *role*.

(2) We rely on background knowledge collected from weak and noisy evidence that is available in existing resources. We have created a dataset of positive examples from descriptions of biological terms from the MeSH and GO databases related to transcription factors. The experiments have shown that although not ideal, this dataset can be used as noisy positive training data.

(3) Feature selection is one of the most important issues in an ML approach. Most of existing approaches rely on weighted word-based features. We have used biological features (such as protein/gene names, molecular interaction words, and TF-related terms) and have shown that these features provide at least comparable performance with a significant reduction of the feature space.

## Conclusions

We have presented a text-classification approach to automatically locate TF-related sentence contexts, in order to build a starting point for literature-based curation of transcription factor databases. The results are highly encouraging, with F-measure well above 90%. The extraction approach is built around an ML-based architecture, with a dedicated feature model based on specific biological features relevant to the task. We have investigated three different ML methods, and also presented a two-stage result-merging method that has been used to combine the results from both different types of machine-learning algorithms and the different training datasets.

Our initial experiments have confirmed that reasonable training data can be obtained from existing resources, namely, MeSH and GO TF-related data. The testing results on the FlyTF data were encouraging, and strongly confirmed our assumptions that TF-related MeSH and GO term definitions are useful for the detection of TF-related contexts, but that real-world positive data (e.g. from FlyTF) are needed to improve recall. Another interesting finding from our experiments is that we have not been able to confirm strong similarity between TF and PPI contexts as expected. By using PPI data as negative examples for the TF-related sentence extraction, we were generally able to obtain comparable if not more accurate results when compared to negative data obtained from non-protein-description data (NonPF).

The results reported here show that the proposed approach is capable of accurately identifying TF-related information from text. However, a number of interesting issues remain to be resolved. The first issue is related to distinguishing transcription factors from other proteins in a TF-related context in which two or more gene and protein names co-occur together. A possible solution is to make use of syntactic relations, combined with biological feature terms to judge the likelihood of a protein being a transcription factor. In addition, FlyTF data, which is treated as an important positive TF example dataset used for classification, is an organism-specific corpus. It is likely that it does not cover all TF-related features for various organisms. Therefore, an analysis of a more diverse TF data for the identification of transcription factors is needed.

## Competing interests

The authors declare that they have no competing interests.

## Authors' contributions

HY designed and implemented the system, performed experiments and evaluated the results. GN motivated and coordinated the study, and helped with the interpretation of the results. JAK participated in the conceptual design and machine learning. HY drafted the first version of the manuscript, and GN prepared the final manuscript, which all authors read and approved.
